# Investigation of dissipation kinetics and half-lives of fipronil and thiamethoxam in soil under various conditions using experimental modeling design by Minitab software

**DOI:** 10.1038/s41598-024-56083-5

**Published:** 2024-03-08

**Authors:** Ahmed F. El-Aswad, Abdallah E. Mohamed, Mohamed R. Fouad

**Affiliations:** 1 Department of Pesticide Chemistry and Technology, Faculty of Agriculture, Alexandria University, Aflaton St., El-Shatby, Alexandria, 21545 Egypt; 2 Land and Water Technologies Department, Arid Lands Cultivation Research Institute, City of Scientific Research and Technological Applications (SRTA-City), New Borg El-Arab, Alexandria, 21934 Egypt

**Keywords:** Fipronil, Thiamethoxam, Soil, Dissipation, Half-life, HPLC, Minitab software, Ecology, Plant sciences, Environmental sciences, Chemistry

## Abstract

To determine the extent of pesticide buildup and their environmental contamination, the environmental half-lives of pesticides are examined. The influence of the factors affecting the half-lives of fipronil and thiamethoxam including soil type, sterilization, temperature, and time and their interactions was studied using experimental modeling design by Minitab software. Based on the dissipation kinetics data, fipronil concentrations reduced gradually over 60 days while thiamethoxam concentrations decreased strongly. Also, fipronil and thiamethoxam dissipated more rapidly in calcareous soil than in alluvial soil. Thiamethoxam, however, disappeared more rapidly than fipronil in all treatments. Incubation at 50 °C leads to rapid the pesticide degradation. For prediction of the dissipation rate, model 5 was found to be the best fit, Residue of insecticide (%) = 15.466 − 11.793 Pesticide − 1.579 Soil type + 0.566 Sterilization − 3.120 Temperature, R^2^ = 0.94 and s = 3.80. Also, the predicted DT_50_ values were calculated by a model, DT_50_ (day) = 20.20 − 0.30 Pesticide − 7.97 Soil Type + 0.07 Sterilization − 2.04 Temperature. The shortest experimental and predicted DT_50_ values were obtained from treatment of thiamethoxam at 50 °C in calcareous soil either sterilized (7.36 and 9.96 days) or non-sterilized (5.92 and 9.82 days), respectively. The experimental DT_50_ values of fipronil and thiamethoxam ranged from 5.92 to 59.95 days while, the modeled values ranged from 9.82 to 30.58 days. According to the contour plot and response surface plot, temperature and sterilization were the main factors affecting the half-lives of fipronil and thiamethoxam. The DT_50_ values of fipronil and thiamethoxam increased in alluvial soil and soil with low temperature. In general, there is a high agreement between the experimental results and the modeled results.

## Introduction

Pesticides are chemical compounds used to control various pests. They include herbicides, insecticides, fungicides, nematicides, rodenticides, molluscicides and plant growth regulators^1,2^. Pesticide use has expanded extensively in recent years, resulting in environmental damage, particularly water and soil contamination. The scientific community has been working hard to come up with creative approaches to reduce pesticide pollution^3^. The environmental pollution by pesticides is correlated with pesticide persistence. When a pesticide breaks down it forms new chemicals that may be more or less toxic than the original chemical. Generally, pesticides are broken until only carbon dioxide, water, and minerals are left^4^.

When estimating whether pesticides tend to accumulate in the environment, environmental half-life must be considered^5^. The half-life (DT_50_) of a pesticide can be defined as the time required to reduce a certain amount of a pesticide by half. Half-lives of compounds are commonly reported as time ranges. The concept of half-life only applies to organic compounds. In the environment, the pesticide concentration decreases through breakdown or dissipation. The transformation rate of pesticides is commonly estimated using first-order kinetics, that the transformation rate is independent of the initial pesticide concentration^6,7^. Half-life of pesticide can vary due to different factors and environmental conditions, including soil type, microbial populations and activity, soil moisture, and temperature^8,9^. Therefore, to control the conditions, the half-lives are determined in a laboratory^6^. Accordingly, pesticides can be divided into three categories based on their half-lives in order to estimate persistence. Non-persistent pesticides that have half-lives less than 16 days, moderate (16–59 days), and persistent pesticides that have half-lives greater than 60 days. In general, persistent, and non-persistent pesticides can have drawbacks. Persistent pesticides may contaminate surface and ground water, animals, and plants. Also, the non-persistent pesticides would likely have to repeat applications, increasing the risk of exposure to animals, plants, and people^5,8^.

Soil, an important component of the environment, acts as a sink for the majority of pesticides used in agriculture^10–12^. Following their reach to the soil, pesticides undergo a variety of degradation and transformation processes^13^. These processes depend on several physicochemical factors, including adsorption/desorption, movement, plant uptake, volatilization, and decomposition^12,14^. Therefore, refined knowledge of the physical and chemical properties of pesticides are necessary to determine the behavior and impact of pesticide decomposition in environment^3^. The mechanisms of the degradation/transformation processes include microbial degradation, photolysis, and chemical hydrolysis^10,13^.

The use of synthetic insecticides, herbicides and fungicides contributed greatly to pest control and improvement of quantity and quality the agricultural output. Ideally a pesticide must be lethal to the targeted pests and safe to non-target species. Unfortunately, this is not the case. The rampant and random use of pesticides has played havoc with humans, environment, and other life forms. Fipronil, a phenyl pyrazole family is a relatively new broad-spectrum insecticide. It is applied via soil, foliar, bait, or seed treatment against a wide range of crop pests^15–17^. Fipronil is registered for non-agricultural as well as agricultural use in many countries. It is widely applied to control thrips, termites, and click beetles on various crops such as rice, cotton, maize, vegetables, and fruits^15,17^. Also, it is used for the eradication of fleas, ticks, and fire ants. However, fipronil is highly toxic to aquatic species^18^. Fipronil acts by blocking the γ-aminobutyric acid (GABA)-regulated chloride channel. The use of fipronil was increased due to many reasons, like its lower application rate compared to conventional pesticides, its environmental safety, its effective against insects that resistant to insecticides whether organophosphate, carbamate, and pyrethroid, and the recent restriction on organophosphate insecticides as well as the bans on organochlorines^15,18^. Half-life of fipronil in soil varies widely, from 3 days to 7 months depending on temperature, moisture content, sterilization, compound formulation, and soil composition^18–20^. In sandy loam and organic matter-rich soil, fipronil degradation was significantly accelerated with the increasing of temperature^21^. Also, at 25 °C, the half-life was 9.72 days in non-sterile clay loam soil compared to 33.51 days in the sterile soil^17^. In addition, among the factors affecting pesticide half-lives, the exposure media. Fipronil dissipated more rapidly in peanut seedlings (1 day half-life) compared to in soil (more than 1 month half-life)^22^.

Thiamethoxam, a second-generation neonicotinoid insecticide, is currently applied widely for foliar, soil, and seed treatment^23,24^. It is effective against insects that damage a variety of crops by sucking and eating like aphids, thrips, whiteflies, plant hoppers, and beetles in rice, maize, cotton, and vegetables^24,25^. It is classified by the EPA as toxicity category III in acute oral and dermal studies. Categorized as a neurotoxin, thiamethoxam acts as an agonist of the nicotinic acetylcholine receptors (nAChR)^26^. In male bumblebees, neonicotinoids have been shown to reduce sperm viability and living sperm quality^23^. The DT_50_ value of thiamethoxam was 4.4 days in green tobacco leaves, 4 days in tomato crops and 18.5 days in soil^26^. Thiamethoxam had DT_50_ of 15.0 days in silty clay loam soil, 16.91 days in sandy clay loam soil, and 20.1 days in loam soil^27,28^. Moreover, according to moisture regimes, DT_50_ ranges from 46.3 to 301.0 days^24^.

Minitab software was created in 1972 at Penn State University to accelerate calculations. Recently, Minitab is widely used in various purposes including design of experiments, and statistical analysis of the results^29,30^. All components of an experiment may be varied simultaneously, systematically, and effectively with statistical designs. During parameter optimization, one parameter is varied while the other parameters are fixed at a single constant level. The benefit of a design by Minitab software is the possibility to examine both the effects of individual components as well as the combined effects of multiple factors and their interactions^31,32^. The effect of temperature and relative humidity on the degradation profiles of some pesticides was established quantitative models for prediction purpose. The quantitative model obtained using Minitab software allows the prediction of the pesticide residual level^33^. Multivariate study of pesticide residues in fruits and vegetables was carried out using Minitab statistical software. It was used to determine the significance of each factor by Pareto chart followed by optimization of these significant factors using central composite design^34^. Dissipation of chlorantraniliprole in soil was estimated under different conditions that were designed by Minitab software^30^.

Therefore, the current investigation was undertaken with the objective of studying the dissipation behavior and half-lives estimation of fipronil and thiamethoxam insecticides under different laboratory conditions that were designed by Minitab software. Different variables were pesticide type (fipronil and thiamethoxam), soil type (alluvial and calcareous soil), temperature (25 and 50 °C), sterilization (sterile and non-sterile soil), incubation time (up to 60 days). In general, modeling studies were used to develop appropriate mathematical models that could predict dissipation half-lives of insecticides tested under different conditions.

## Materials and methods

### Chemicals

Fipronil, (±)-5-amino-1-(2,6-dichloro-α,α,α-trifluoro-p-tolyl)-4-trifluoromethyl-sulfinylpyrazole-3-carbonitrile and thiamethoxam, (EZ)-3-[(2-chlorothiazol-5-yl) methyl]-5-methyl-*N*-nitro-1,3,5-oxadiazinan-4-imine, 99.9%, purity were supplied by Shandong Chuangying Chemical Co. and Shandong SanYoung Industry Co., Ltd, respectively. The chemical structures and the properties are given in Table 1. Fipronil has a low water solubility, high octanol–water partition coefficient and very poor vapor pressure. Thiamethoxam has high water solubility, low octanol water partition coefficient and low vapor pressure. Fipronil and thiamethoxam are relatively new broad-spectrum insecticides. Currently, their use was increased. They are environmentally safe and effective against insects that are resistant to different insecticide groups organophosphate, carbamate, and pyrethroid. They are applied widely for soil, foliar, and seed treatment. Hence, they reach easily to soil. Half-life of fipronil and thiamethoxam in soil varies widely, from few days to few months depending on soil composition and temperature. Fipronil and thiamethoxam can be harmful to non-intended targets and beneficial insects like honeybees so you will need to be careful where you apply it. Anhydrous sodium sulfate (Na_2_SO_4_), sodium hydroxide (NaOH), also commercial solvents including acetonitrile, acetone, formic acid, and dimethyl formamide were purchased from Al Gomhoria Chemical Co., Alexandria, Egypt. Solvents HPLC-grade methanol, dichloromethane, and acetonitrile were purchased from Sigma Aldrich Co. (Spruce Street, Louis., MO, USA).

**Table 1:** Chemical structure and properties of tested insecticides.

Properties	Fipronil	Thiamethoxam
Chemical structure		
Chemical formula	C_12_H_4_Cl_2_F_6_N_4_OS	C_8_H_10_ClN_5_O_3_S
CAS Number	120068-37-3	153719-23-4
Molar mass	437.14 g mol^−1^	291.71 g mol^−1^
Group	Phenylpyrazole	Neonicotinoid
Solubility in water at 20 °C	0.0024 g L^−1^	4.1 g L^−1^
Octanol–Water Partition Coefficient (Kow)	1.00 × 10^4^	0.74
Vapor pressure (mmHg) at 25 °C	2.80 × 10^−9^	4.95 × 10^−11^
Usage	It is used for controlling multiple species of thrips on a broad range of crops by foliar, soil or seed treatment. Control of corn rootworm, wireworms, and termites by soil treatment in maize. Soil treatment rates range from 100–200 g/ha. Foliar application rates 10–80 g/ha	It is used for controlling aphids, whiteflies, thrips, ricehoppers, Colorado potato beetle, flea beetles, wireworms, ground beetles, and leaf miners at 10 to 200 g/ha. It is applied by foliar, soil, and seed treatments. Also, it is used for control of flies in animal and public health

### Tested soils

Two common soil types alluvial and calcareous that representative the Egyptian soils were used in this study. The soil samples were collected from the surface layer (0–20 cm) from different locations that have no history with pesticides. The alluvial soil was collected from the Agricultural Research Station, Abis farm of the Faculty of Agriculture, University of Alexandria, and the calcareous soil was collected from the Elnahda region, Elamria, Alexandria Governorate. The physical and chemical properties were determined^35,36^ at the Department of Soil and Water Sciences, Faculty of Agriculture, University of Alexandria, the data are presented in Table 2. Soil samples were air-dried, ground, and passed through a 2-mm sieve. The soil texture was determined by the hydrometer method. The pH of the soil was measured in the presence of 0.01 M calcium chloride (CaCl_2_) at 1:2 (w/w), soil: solution slurry. The organic matter (OM) and organic carbon (OC) contents were determined by dichromate oxidation according to the Walkley–Black method. Soil analysis indicated that the alluvial soil is clay soil type while the calcareous soil is sandy clay loam soil. The OM content in clay soil (3.4%) was more than twice that in sandy clay loam soil (1.5%). Both soil types of clay and sandy clay loam were alkaline soil. The calcareous soil was the highest in soluble cations and anions, consequently electrical conductivity.

**Table 2:** Physical and chemical properties of tested soils.

Soil type	Alluvial soil	Calcareous soil
Texture class	Clay	Sandy clay loam
Water holding capacity (mL g^−1^)	0.46	0.38
EC (ds m^−1^)	1.3	5.0
Soil pH	8.3	8.2
Organic matter content (%)	3.4	1.5
Soluble cations conc. (meq L^−1^)	18.7	48.3
Soluble anions conc. (meq L^−1^)	13.3	50.4

### Experimental design using Minitab software

The dissipation experiment of tested insecticides in soil under different conditions was designed using response surface methodology by Minitab software (Minitab Inc., USA)^30,37^. The design included many selected variables to provide a wide range of possibilities. The variables were pesticide type (fipronil and thiamethoxam), soil type (alluvial and calcareous), sterilization (with and without), temperature of incubation (25 and 50 °C), as well as the time intervals. Temperature of 25 °C was chosen in this study because it is a normal temperature throughout the year in Egypt. While 50 °C was selected because some locations in particular at upper Egypt are very hot and the temperature may be exceeded 45 °C. Moreover, with climatic change, the temperature may rise to very high levels. Using a two-level factorial design at zero center point, 16 treatments described in Table 3 were carried out.

**Table 3:** Experimental design of fipronil and thiamethoxam dissipation in soil using Minitab software.

Treatment	Pesticide	Soil Type	Sterilization	Temperature (°C)
1	Fipronil	Alluvial	Sterilized	25
2	Thiamethoxam	Alluvial	Non-sterilized	25
3	Fipronil	Calcareous	Sterilized	25
4	Fipronil	Calcareous	Non-sterilized	50
5	Fipronil	Calcareous	Non-sterilized	25
6	Thiamethoxam	Alluvial	Non-sterilized	50
7	Fipronil	Alluvial	Non-sterilized	25
8	Fipronil	Alluvial	Sterilized	50
9	Thiamethoxam	Calcareous	Sterilized	50
10	Thiamethoxam	Calcareous	Non-sterilized	50
11	Thiamethoxam	Alluvial	Sterilized	50
12	Fipronil	Calcareous	Sterilized	50
13	Fipronil	Alluvial	Non-sterilized	50
14	Thiamethoxam	Calcareous	Non-sterilized	25
15	Thiamethoxam	Alluvial	Sterilized	25
16	Thiamethoxam	Calcareous	Sterilized	25

### Soil treatment

The soil samples were autoclaved at 120 °C at 15 psi for 15 min to destroy the microbes responsible for the degradation of pesticides before initiating the experiment^15^. A weight of 100 g of soil (alluvial or calcareous) was placed in 350-mL glass bottle and treated with fipronil or thiamethoxam (100 μg a.i. g^−1^ soil). Five replicates were carried out for each treatment. Distilled water was used to provide 60% of the water holding capacity of the soil. Based on the Minitab design, the bottles were incubated throughout the experimental period^30^.

### Determination of fipronil and thiamethoxam in soil by HPLC

#### Preparation of standard solutions

Individual standard solutions (1000 µg mL^−1^) of fipronil and thiamethoxam were prepared by dissolving 10,000 µg of fipronil or thiamethoxam into a volumetric flask, then the volume was completed to 10 mL with acetonitrile-HPLC grade and stored at 4 °C in the dark. Working solutions of tested compounds were prepared by dilution to reach the required final concentration (100 µg mL^−1^).

#### HPLC method validation

**Figure 1:**
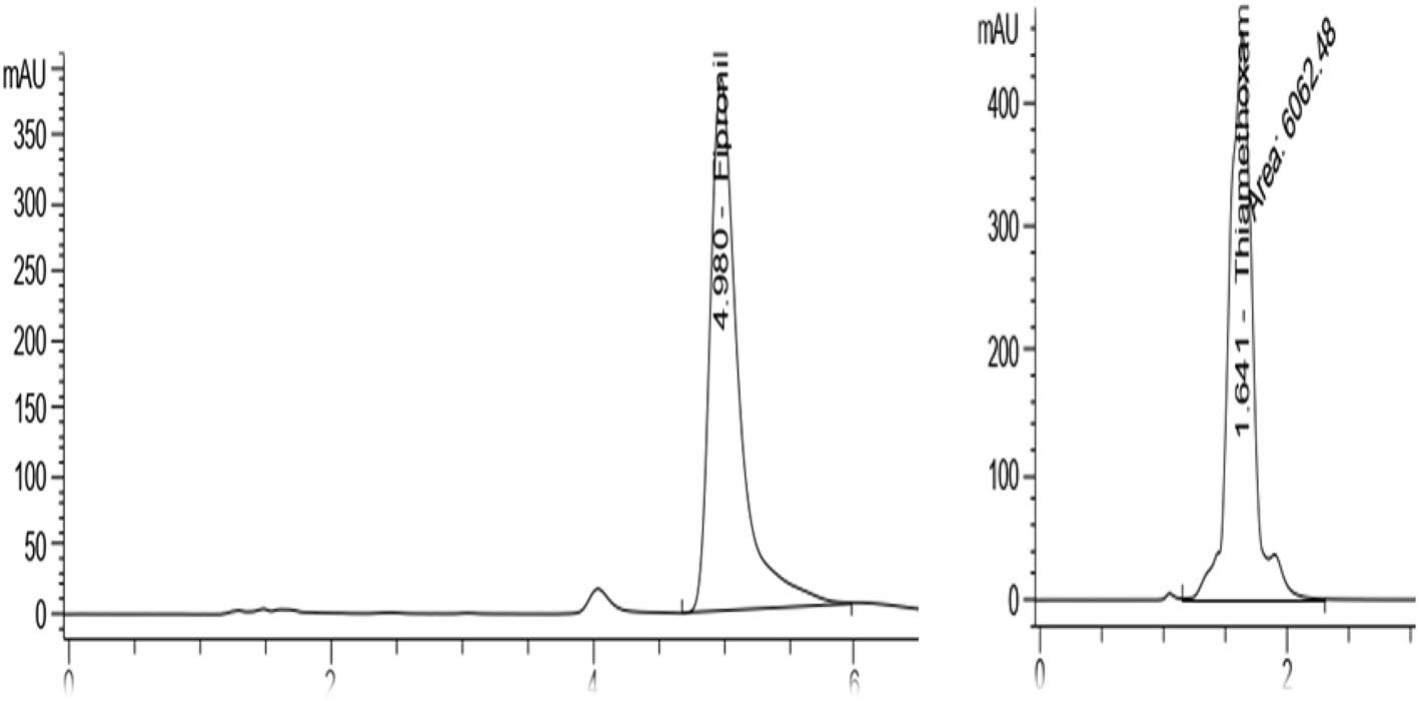
The HPLC chromatograms of fipronil (left) and thiamethoxam (right) showing the retention times.

The development and validation method for determination of fipronil and thiamethoxam residues were performed on Agilent 1260 HPLC Infinity system (Germany) equipped with an Agilent variable wavelength ultraviolet detector (VWD). The linearity of the instrument for fipronil and thiamethoxam was confirmed by plotting the chromatographic calibration curves. The retention time was found to be 4.980 min for fipronil and 1.641 min for thiamethoxam. The limits of detection (LOD) and quantification (LOQ) for fipronil were 0.021 and 0.063 μg g^−1^, respectively. These values are in agreement with those obtained by^38^. The HPLC chromatograms of fipronil and thiamethoxam are shown in Fig. 1.

#### Extraction and purification of tested pesticides

The homogenized samples (5 g) were weighed into a 25-mL centrifuge tube and extracted with 10 mL of acetonitrile-0.1% formic acid: dimethyl formamide (8:2). The samples were grinded in a mortar with 3 g anhydrous sodium sulfate (Na_2_SO_4_) for 5 min. They were placed in glass tubes and shaken in a water bath at 35 °C for 30 min. Then the samples were centrifuged at 6000 rpm for 5 min and filtered through Whatman filter paper No. 1. Finally, the prepared samples were filtered through a 0.22-mm nylon syringe filter and transferred to an autosampler vial prior to HPLC residue analysis^39^.

#### Determination by HPLC

The quantification of fipronil and thiamethoxam was determined by an Agilent 1260 HPLC Infinity system (Germany). Twenty microliters of each sample extract were injected onto the HPLC column using the autosampler apparatus with a 100 μL sample loop. Separation was performed on XDB ZORBAX Eclips Plus C18 column (250 × 4.6 mm id, 5 µm particle size). The mobile phase composition was water and acetonitrile (60:40 v/v) with a flow rate of 1 mL min^−1^. The Column temperature was 30 °C. The detection wavelength was 270 nm for fipronil and 255 nm for thiamethoxam. The data were managed using HP Chemstation software.

#### Recovery assay

Untreated soil samples were homogenized before being spiked with standard solutions of fipronil and thiamethoxam at three fortification levels 5, 10, and 25 µg g^−1^ soil. The samples were processed according to the above procedure. The averages of recovery for different fortifications of fipronil were 98.0 ± 1.8% and 99.7 ± 0.2% and that of thiamethoxam were 99.6 ± 0.4% and 96.5 ± 3.0% in alluvial and calcareous soil, respectively. The obtained results were corrected according to the recovery rates.

### Statistical analysis

Minitab software (Minitab^®^ 16.1.0.0. 2019 Inc.) was used to design the experiments as well as the calculation of means and standard errors. Various plots (scatter, histograms and normal probability) of insecticide residues were used to examine model adequacy checks.

## Results and discussion

### Dissipation of fipronil and thiamethoxam in soil

**Figure 2:**
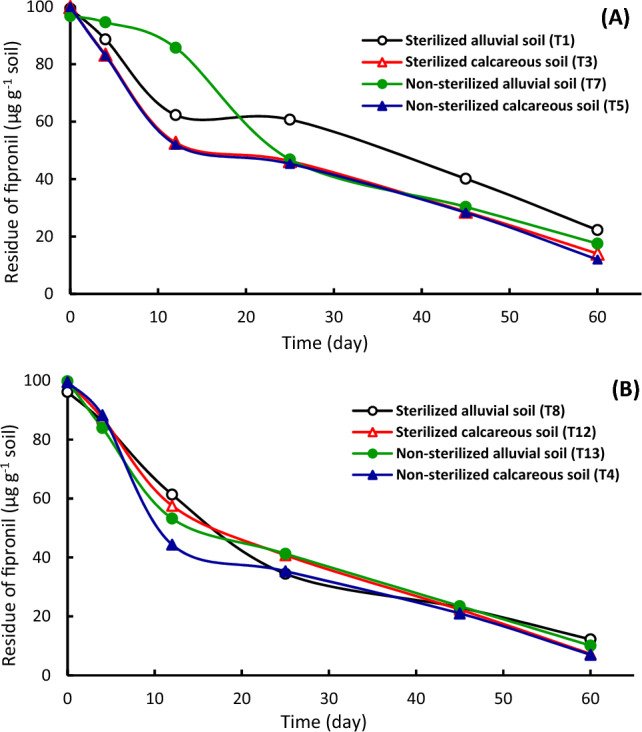
Dissipation of fipronil in sterilized and non-sterilized alluvial and calcareous soil. (**A**) At 25 °C, (**B**) at 50 °C.

**Figure 3:**
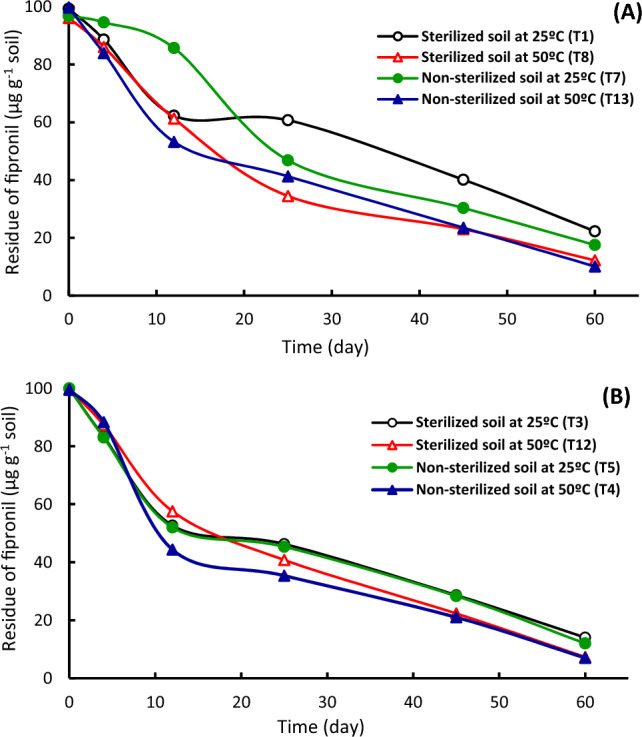
Dissipation of fipronil in sterilized and non-sterilized soil at 25 and 50 °C. (**A**) Alluvial soil, (**B**) Calcareous soil.

**Figure 4:**
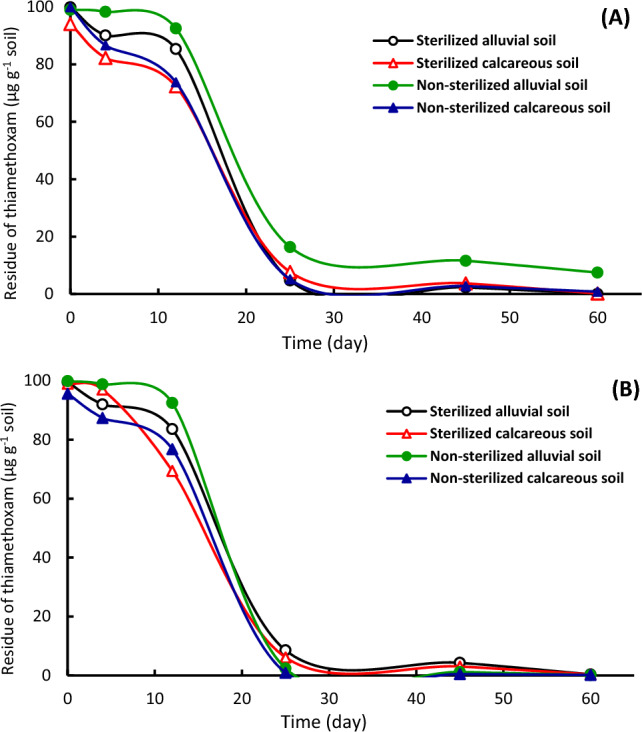
Dissipation of thiamethoxam in sterilized and non-sterilized alluvial and calcareous soil. (**A**) At 25 °C, (**B**) at 50 °C.

**Figure 5:**
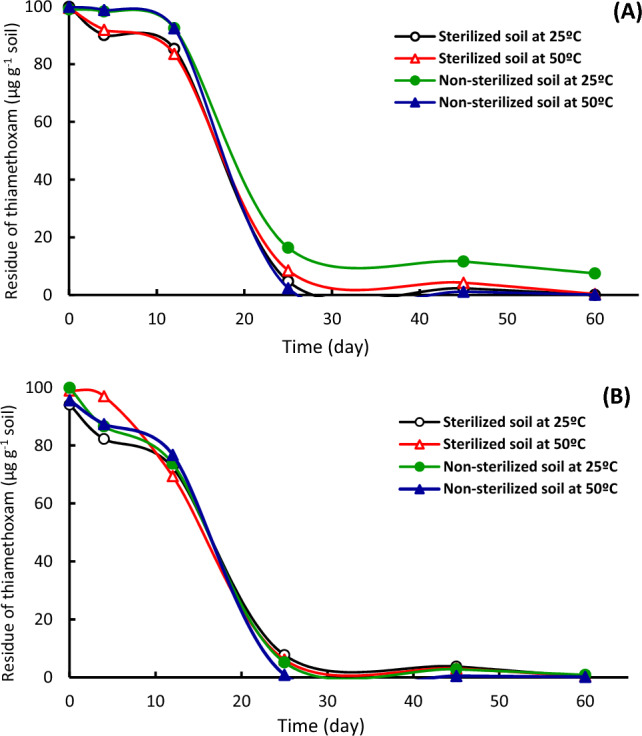
Dissipation of thiamethoxam in sterilized and non-sterilized soil at 25 and 50 °C. (**A**) Alluvial soil, (**B**) Calcareous soil.

The effect of soil type, soil sterilization and temperature on the dissipation kinetics of fipronil and thiamethoxam was investigated using laboratory experiments. The disappearance curves of fipronil (Figs. 2, 3) and thiamethoxam (Figs. 4, 5) in alluvial and calcareous soil at 25 and 50 °C were shown. The disappearance kinetic curves indicated that the dissipation of fipronil was rapid in sterilized and non-sterilized calcareous soil at 25 °C. Also, the dissipation rate of fipronil in non-sterilized alluvial soil increased after the first three weeks that may be as a lag period of the microorganisms in the soil (Fig. 2A). The disappearance curves of fipronil were almost the same in sterilized and non-sterilized conditions in the case of alluvial soil at 50 °C (Fig. 2B) and in calcareous soil at 25 and 50 °C (Fig. 2A,B). Figure 3 shows the effect of temperature on dissipation of fipronil in soil. At 25 °C, the dissipation kinetic of fipronil in non-sterilized alluvial soil was slow then it increased starting from 21st day (Fig. 3A). The dissipation in sterilized and non-sterilized alluvial soil (Fig. 3A) and in sterilized and non-sterilized calcareous soil (Fig. 3B) was rapid at 50 °C compared to that at 25 °C. The decomposition of fipronil under these conditions might be a thermo-chemical breakdown.

Dissipation kinetics of thiamethoxam at different conditions was determined in sterilized and non-sterilized alluvial and calcareous soil at 25 and 50 °C incubation (Figs. 4, 5). Thiamethoxam rapidly disappeared in sterilized and non-sterilized calcareous soil than that in alluvial soil (Fig. 4A,B). This might be due to its low adsorption on calcareous soil. The decline of the disappearance curves was strong from 0 to 28th day in sterilized calcareous and alluvial soil while from the 15th to the 28th day in non-sterilized alluvial soil. The disappearance curves of thiamethoxam were identical at 25 °C in calcareous soil and alluvial soil after 15 days of incubation (Fig. 4A). In non-sterilized alluvial soil at 25 and 50 °C (Fig. 5A), the compound persisted against degradation through the first two weeks, after that it broke down. The dissipation kinetic rate at 25 °C was lower throughout the second month compared to that in non-sterilized alluvial soil at 50 °C (Fig. 5A). The disappearance curves of thiamethoxam were identical at 25 and 50 °C in sterilized alluvial soil (Fig. 5A) and sterilized and non-sterilized calcareous soil (Fig. 5B).

In general, the dissipation kinetics of fipronil and thiamethoxam was rapidly in calcareous soil than in alluvial soil. The higher persistence of fipronil and thiamethoxam in alluvial soil compared to the calcareous soil was attributed to higher adsorption capacity of pesticides to clay particles along with higher organic matter content of alluvial soil. This suggestion is in agreement with that obtained by^15,20,30^. Calcium carbonate content in calcareous soil plays an important role for pesticide behavior in the soil^12^. Also, our results indicated that the disappearance rate of tested pesticides is rapid at 50 °C compared to that at 25 °C. Adsorption of pesticides decreases at high temperature^40^. Adsorption of pesticides on organic matter reduces their availability where they can be persistent^41,42^. The adsorption of pesticides protects compounds against degradation, particularly biodegradation that requires a time interval as a lag period before degradation. Degradation of fipronil has an initial slower rate followed by a faster rate^43^. Degradation of fipronil in soil in the laboratory and field is accelerated by microorganisms^44^, and the lag period is the critical phase^45^. Fipronil in environment can undergo hydrolysis, photolysis, oxidation, or reduction^46^. The rate of its degradation depends on various factors including microbial communities in soil^43,47^. About the impact of sterilization on tested pesticide dissipation in soil, the results verified that non-sterilized soil treatments had higher dissipation than serialized soil. This result is consistent with earlier research for other pesticides, which showed that the pesticide dissipation was more rapid in non-sterilized soil than sterilized soil^30^. While, at 50 °C temperature, no effect of sterilization or adsorption was obtained, suggesting that the dissipation might be via chemical decomposition. Incubation at 50 °C temperatures leads to the rapid degradation of pesticides^20,30^. Moreover, thiamethoxam was more disappear than fipronil in all treatments, which was attributed to high adsorption capacity of fipronil on soil particles and organic matter compared to thiamethoxam^16,25,48^. Also, thiamethoxam has low sorption in soil^49,50^, which suggests availability of the insecticide in soil solution^48,51^. The degradation rate increased with the increase of the organic carbon content in the soil. Also, the moisture content in the soil had a positive effect on the degradation rate^52^.

### Dissipation modeling study of fipronil and thiamethoxam in soil

Results indicated that the variables tested (pesticide type, soil type, sterilization, and temperature) had a significant impact on the dissipation rate of the pesticides. Table 4 displays the outcomes of the models produced by the Minitab software used to create factorial design at various time intervals. Six models were generated by Minitab software, a model for each time interval to study the disappearance of tested pesticides. All models were generated with correlation coefficient (R^2^ from 0.48–0.95) and s value (2.96–7.92). The highest fit model for prediction of the dissipation study was model 5 (Residue of insecticide (%) = 15.466 − 11.793 Pesticide − 1.579 Soil type + 0.566 Sterilization − 3.120 Temperature), R^2^ = 0.94 and s = 3.80, followed by model 4 (Residue % = 25.26 − 18.76 Pesticide − 1.67 Soil type + 1.05 Sterilization − 4.03 Temperature), R^2^ = 0.95 and s = 5.39 (Table 4). While the completely invalid model was model 2 (Residue % = 89.35 + 2.20 Pesticide − 2.20 Soil type − 0.76 Sterilization + 0.74 Temperature), R^2^ = 0.48 and s = 4.80.

**Table 4:** Proposed models obtained from Minitab software for tested pesticides in soil at different time intervals.

Model no.	Time (day)	Models of insecticide residues in soil Residue of insecticide (%) =	S	R^2^
1	0	92.81 − 3.973 Pesticide − 2.376 Soil type − 0.065 Sterilization − 1.924 Temperature	3.01	0.81
2	4	89.35 + 2.20 Pesticide − 2.20 Soil type − 0.76 Sterilization + 0.74 Temperature	4.80	0.48
3	12	70.19 + 10.58 Pesticide − 6.87 Soil type − 1.16 Sterilization − 2.85 Temperature	7.92	0.80
4	25	25.26 − 18.76 Pesticide − 1.67 Soil type + 1.05 Sterilization − 4.03 Temperature	5.39	0.95
5	45	15.466 − 11.793 Pesticide − 1.579 Soil type + 0.566 Sterilization − 3.120 Temperature	3.80	0.94
6	60	7.007 − 5.801 Pesticide − 1.764 Soil type + 0.097 Sterilization − 2.302 Temperature	2.96	0.88

### Experimental and modeled residue data of fipronil and thiamethoxam in soil

The pesticide residues of different treatments at various time intervals which were measured experimentally by HPLC and determined theoretically by Minitab software are presented in Table 5. The difference between experimental values and modeled values of pesticide residues was very narrow at 25th and 45th time intervals. The lowest difference value was approximately zero in T3 (fipronil, calcareous soil, sterilization, 25 °C) and T5 (fipronil, calcareous soil, non-sterilization, 25 °C). The residue percentages were 47.41 and 47.43% on the 25th day and 29.43 and 29.37% at 45th day in T3 and that were 45.33 and 45.33% at 25th day and 28.33 and 28.23% at 45th day in T5 for experimental and modeled determination, respectively. Whereas the highest difference value was lower than 10 in T1 (fipronil, alluvial soil, sterilization, 25 °C), the residue percentages for experimental and modeled determination were 60.72 and 50.77% at 25th day and 40.13 and 32.52% on the 45th day, respectively. Pesticide residues were detected in all treatment samples up to 60 days after incubation, with a gradual reduction in concentration of fipronil over time whereas, a strong decline in concentration of thiamethoxam. In treatment of T1 and T13, the concentration of fipronil was gradually reduced from 62.31 to 60.72% and from 53.21 to 41.27% throughout the interval between the day of 12th to 25th, respectively. While, in treatment of T2 and T14, the concentration of thiamethoxam was strongly decreased from 92.51 to 16.40% at 12th day and from 73.75 to 5.15 at 25th day, respectively. More than 7% for fipronil while lower than 1% for thiamethoxam except T2 (alluvial soil, non-sterilization, 25 °C) were detected at 60th day incubation of all treatments. According to the average of pesticide residue values, the different treatments could be divided into three categories. First category has difference values between experimental and modeled residues (0–< 2), including T4, T5, T14, T16. The differences (2–< 3) were found in T1, T3, T7, T8, 10, T11, T12 (2nd category). Third category including T2, T6, T9, T13, T15, the difference between their experimental and modeled data ranged from 3 to < 5 (Table 5).

**Table 5:** Residue percentages of fipronil and thiamethoxam in soil at different time intervals determined experimentally by HPLC and modeled by Minitab software.

Treatment	Determination	Time (day)						Average
		0	4	12	25	45	60	
T1	Experimental	99.33	88.67	62.31	60.72	40.13	22.27	62.24
	Modeled	101.04	87.85	68.17	50.77	32.52	16.97	59.55
T2	Experimental	98.99	98.28	92.51	16.40	11.62	7.51	54.22
	Modeled	93.22	93.77	91.65	11.15	7.81	5.18	50.46
T3	Experimental	99.87	85.33	60.34	47.41	29.43	14.03	56.07
	Modeled	96.28	83.45	54.43	47.43	29.37	13.44	54.07
T4	Experimental	99.49	88.28	44.35	35.36	21.02	7.00	49.25
	Modeled	92.53	86.45	51.05	37.27	21.99	8.65	49.66
T5	Experimental	99.93	83.00	52.00	45.33	28.33	12.00	53.43
	Modeled	96.41	84.97	56.75	45.33	28.23	13.25	54.16
T6	Experimental	99.90	98.78	92.47	2.40	1.16	0.13	49.14
	Modeled	89.34	95.25	85.95	3.09	1.57	0.57	45.96
T7	Experimental	96.79	94.60	85.72	46.85	30.34	17.53	61.97
	Modeled	101.17	89.37	70.49	48.67	31.39	16.78	59.64
T8	Experimental	96.13	86.07	61.33	34.46	23.00	12.21	52.20
	Modeled	97.15	89.33	62.47	42.71	26.28	12.37	55.05
T9	Experimental	98.98	97.00	69.41	6.12	3.03	0.36	45.82
	Modeled	84.45	89.33	69.89	1.85	0.00	0.00	40.92
T10	Experimental	95.60	87.38	76.78	0.89	0.45	0.17	43.55
	Modeled	84.58	90.85	72.21	0.00	0.00	0.00	41.27
T11	Experimental	99.50	91.96	83.59	8.53	4.29	0.35	48.04
	Modeled	89.21	93.73	83.63	5.19	2.70	0.77	45.87
T12	Experimental	99.67	87.30	57.57	40.78	22.32	7.32	52.49
	Modeled	92.40	84.93	48.73	39.37	23.13	8.84	49.57
T13	Experimental	99.78	83.95	53.21	41.27	23.50	10.10	51.97
	Modeled	97.28	90.85	64.79	40.61	25.15	12.17	55.14
T14	Experimental	99.90	86.61	73.75	5.15	2.78	0.84	44.84
	Modeled	88.47	89.37	77.91	7.81	4.65	1.65	44.98
T15	Experimental	99.90	90.10	85.33	4.77	2.32	0.07	47.08
	Modeled	93.09	92.25	89.33	13.25	8.94	5.37	50.37
T16	Experimental	94.08	82.25	72.34	7.70	3.74	0.22	43.39
	Modeled	88.34	87.85	75.59	9.91	5.78	1.84	44.88

### Experimental and modeled half-life (DT_50_) values of fipronil and thiamethoxam in soil

The residues of fipronil and thiamethoxam were calculated by first-order exponential decay equation (${C}_{t}={C}_{0}{e}^{-kt}$). According to the experimental data, K was calculated from K = [2.303/t2 − t1] log [C1/C2] and DT50 values of tested pesticides were calculated from DT50 = 0.6932/K. Dissipation of fipronil and thiamethoxam followed first-order kinetics with good fit^53,54^. Also, the predicted DT50 values of tested pesticides in soil at different conditions were calculated by a model produced from Minitab software, the model as follows,

$$ {\text{DT}}_{50} \;({\text{day}}) = 20.20 \, - 0.30\;{\text{Pesticide }} - 7.97\;{\text{Soil}}\;{\text{Type}} + 0.07\;{\text{Sterilization }} - 2.04\;{\text{Temperature}}. $$

The linear equations for different treatments and values of correlation coefficient (R^2^), constant (K), and DT_50_ of tested pesticides in soil were summarized in Table 6. All equations of treatments have high correlation coefficient (R^2^ from 0.89–0.99). The constant K values for pesticide disappearance kinetics in soil ranged from 0.0116 to 0.1172. The highest K value (0.1172) was obtained from T10, whereas the lowest value was shown in T6 (0.0116) followed by T15 (0.0118).

**Table 6:** Residue kinetics of tested insecticides in soil fitted in the first-order kinetic model.

Treatments	Linear equation	R^2^	K	DT_50_ (day)	
				Experimental	Modeled
T1	y = − 0.0225x + 4.5674	0.96	0.0225	30.80	30.58
T2	y = − 0.0478x + 4.6582	0.91	0.0478	14.50	29.84
T3	y = − 0.0302x + 4.5740	0.98	0.0302	22.95	14.64
T4	y = − 0.0403x + 4.5517	0.96	0.0403	17.20	10.42
T5	y = − 0.0316x + 4.5389	0.96	0.0316	21.93	14.50
T6	y = − 0.0116x + 4.9313	0.94	0.0116	59.95	25.76
T7	y = − 0.0292x + 4.6600	0.99	0.0292	23.74	30.44
T8	y = − 0.0336x + 4.5588	0.99	0.0336	20.63	26.50
T9	y = − 0.0942x + 4.8451	0.96	0.0942	7.36	9.96
T10	y = − 0.1172x + 4.6346	0.89	0.1172	5.92	9.82
T11	y = − 0.0925x + 4.9345	0.95	0.0925	7.49	25.90
T12	y = − 0.0403x + 4.6357	0.97	0.0403	17.20	10.56
T13	y = − 0.0353x + 4.5588	0.98	0.0353	19.63	26.36
T14	y = − 0.0839x + 4.6844	0.94	0.0839	8.26	13.90
T15	y = − 0.0118x + 5.0765	0.94	0.0118	58.73	29.98
T16	y = − 0.0977x + 4.8871	0.95	0.0977	7.10	14.04

The calculated data showed that T6 (thiamethoxam, alluvial soil, non-sterilized, 50 °C), and T15 (thiamethoxam, alluvial soil, sterilized, 25 °C) have highest DT_50_ values 59.95 and 58.73 days, respectively. While T10 (thiamethoxam, calcareous soil, non-sterilized, 25 °C), T16 (thiamethoxam, calcareous soil, sterilized, 25 °C), T9 (thiamethoxam, calcareous soil, sterilized, 50 °C), and T11 (thiamethoxam, alluvial soil, sterilized, 50 °C) have lowest DT_50_ values 5.92, 7.10, 7.36, and 7.49 days, respectively. However, the highest modeled DT_50_ values (about 30.5 days) were predicted in T1 (fipronil, alluvial soil, sterilized, 25 °C) and T7 (fipronil, alluvial soil, non-sterilized, 25 °C). It is very interesting to observe that the shortest DT_50_ based on either experimental or predicted data was obtained from T9 (7.36 and 9.96 days) and T10 (5.92 and 9.82 days), respectively. The calculated and predicted DT_50_ values of T1 were almost identical (30.70 ± 0.15 days). In contrast, the calculated DT_50_ values for T6 (59.95 days) and T15 (58.73 days) were about twice the modeled values (25.76 and 29.98 days), respectively.

The results showed that there is a fit between the calculated values and the predicted values. The modeled DT_50_ of these pesticides ranged from 9.82 to 30.58 days and the experimental DT_50_ values ranged from 5.92 to 59.95 days (Table 6). The DT_50_ values of fipronil and thiamethoxam ranged from 17.20–30.80 days and 5.92–59.95 days according to the experimental results, whereas that ranged from 10.42–30.58 days and 9.82–29.98 days according to the modeled data, respectively. The half-life of fipronil was reported to be 30.10 days in sandy loam soil and 37.63 days in clay loam soil^15^. Also, the half-life of fipronil was 132 days under laboratory conditions^44^. In addition, increasing of temperature from 4 to 30 °C reduced the half-life of fipronil in soil from about 3 months to 43 days^21^. Thiamethoxam half-life varied from 46.3 to 301.0 days. The half-life was 46.3–75.3 days under submerged conditions, 91.2–94.1 days under field capacity moisture, and 200.7–301.0 days under dry conditions^24^. Generally, the DT_50_ of pesticide in soil depends on pesticide type, the soil type, sterilization, temperature, pH, type of application, dosage, interval between applications and the environmental conditions^20,30^.

### Contour plots and surface plots of fipronil and thiamethoxam in soil

**Figure 6:**
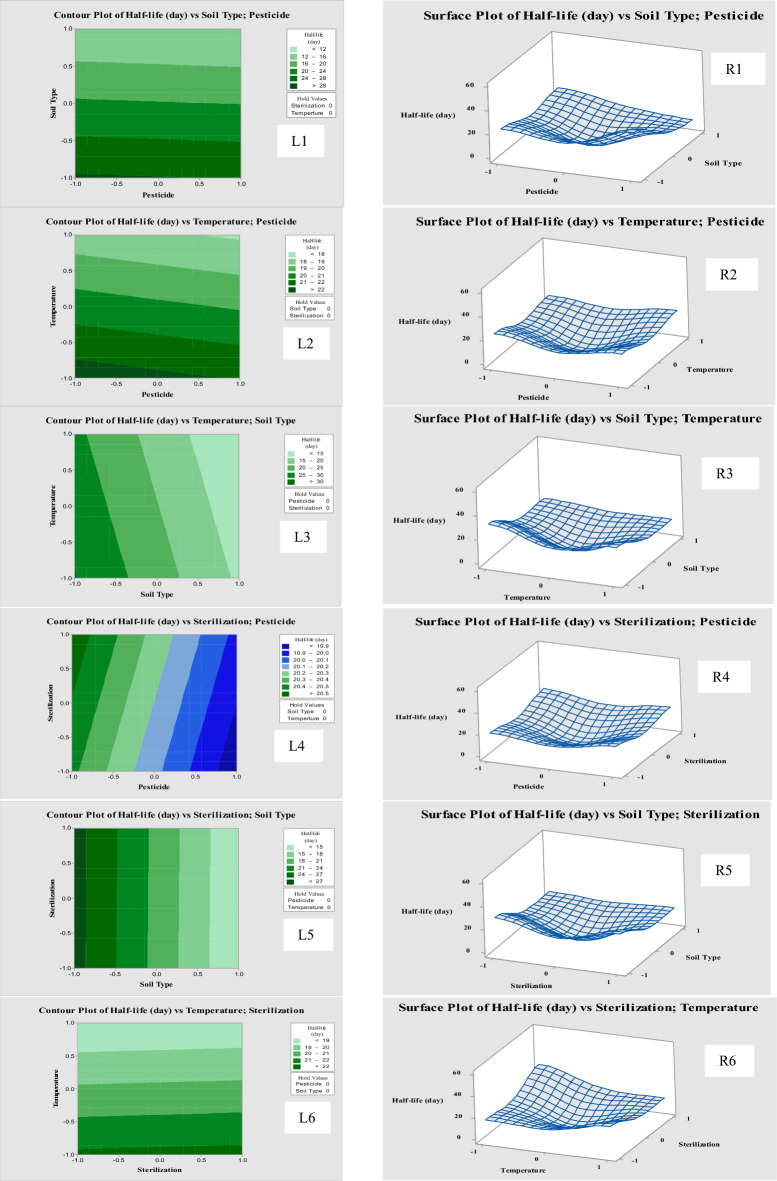
Contour plots (lift, L1–L6) and surface plots (right, R1–R6) of fipronil and thiamethoxam in soil at different conditions depended on predicted DT_50_ model.

The 2D contour plots have been employed to illustrate the effective parameters as designed factors on DT_50_ of tested pesticides in treated soils. Contour plots are drawn as a function of two factors at the same time while all the other factors should be held at fixed levels (at zero level). These plots are useful for examining how influences, both direct and indirect, interact to produce intriguing responses^55^. Figure 6 shows the contour plot for six relations as follows; (1) Pesticide vs. soil type, the value of sterilization and temperature were considered constant at zero fixed level. (2) Pesticide vs. sterilization, the value of soil type and temperature were considered constant at zero fixed level. (3) Pesticide vs. temperature, the value of soil type and sterilization were considered constant at zero fixed level. (4) Soil type vs. sterilization, the value of pesticide and temperature were considered constant at zero fixed level. (5) Soil type vs. temperature, the value of pesticide and sterilization were considered constant at zero fixed level. (6) Sterilization vs. temperature, the value of pesticide and soil type were considered constant at zero fixed level. In general, the contour plot indicates that the DT_50_ values of fipronil and thiamethoxam increased in alluvial soil (Fig. 6, L1), whether at 25 or 50 °C (L3), and whether with sterilization or non-sterilization (L5). Also, it was increased with low temperature (L2) and whether with sterilization or non-sterilization (L6). The DT_50_ value of fipronil increased with sterilization (L4).

The term “response surface methodology” refers to a collection of empirical methods used to assess the relationships between a collection of controlled experimental variables and the measured responses considering one or more predetermined criteria. In the traditional approach to optimization, one parameter is changed at a time while the others are held constant (zero, for instance). In contrast to factorial design, this frequently does not produce the effect of the interaction of numerous parameters. Response surface plots (three-dimensional) are a useful model for studying the effect of several factors influencing responses by varying them simultaneously and carrying out a limited number of experiments^56^. These plots can be easily obtained by calculating using the values taken by one factor where the second varies (from − 1 to + 1) with constraint of a given Y value. The relevant response surface plots can also be used to predict the yield values for varied concentrations of the variables. In the response surface graphic, the surface that is constrained represents the greatest projected yield. The response surface plot obtained (Fig. 6) as a function of pesticides concentration vs. soil type, pesticides concentration vs. sterilization, pesticides concentration vs. temperature, soil type vs. sterilization, soil type vs. temperature, and sterilization vs. temperature, while all other variables are maintained at zero level in each case. An increase in the DT_50_ values were observed in alluvial soil depending on temperature (Fig. 6, R3) and sterilization condition (R5). Also, the DT_50_ values of fipronil and thiamethoxam were increased with sterilization (R4). In addition, the DT_50_ value of fipronil was increased in calcareous soil (R1) and with sterilization condition (R6). Accordingly, the main factors affecting on the half-lives of fipronil and thiamethoxam are temperature and sterilization. These findings support the predicted values and the model's efficacy (three-dimensional). The effectiveness of the experimental variables on the responses can be examined by 3D response surfaces^57^. Therefore, response surface methodology and experimental factorial design can be employed to reduce time, cost, and effort.

Recently, contour plots and response surface plots were used for investigating the effects of different factors on pesticide dissipation behavior. The effect of pH and temperature on the degradation rate of acephate was studied. It was revealed that the optimal conditions for degradation were 36° and pH 6.85^58^. Also, the response-surface-methodology was used in the optimization of distinctive environmental factors such as pH, temperature, agitation-speed, and atrazine-concentration on atrazine degradation^59^. For the pesticide registration in the European Union, model simulations for environmental condition scenarios are used to predict the persistence of pesticides. Scenarios derivation is complicated by uncertainty about fate parameters of pesticides and soil^60^. The interaction of each parameter effect on pesticide degradation was checked by using surface plots and contour plots. 3D graph showed interaction of two parameters on the total amount of pesticide degradation^61^. Similar investigations have been carried out by many researchers for the optimization of various industrial processes. The model determines the relationship between responses and variables and calculates the optimal responses^62^.

## Conclusion

The results made it abundantly evident that the variables chosen for this study had a significant impact on the tested pesticides' dissipation behavior and their half-lives (pesticide type, soil type, sterilization, and temperature). The difference between the experimental values obtained by HPLC and the modeled values produced by the prediction models of pesticide residues was very narrow on the 25th and 45th day. No difference in values was observed in T3 (fipronil, calcareous soil, sterilization, 25 °C), whereas the highest difference value (< 10) was in T1 (fipronil, alluvial soil, sterilization, 25 °C). A gradual decrease in fipronil concentration occurred until reaching about 7%, while a strong decrease in thiamethoxam concentration occurred until reaching less than 1% over time up to 60 days. All linear equations for different treatments have high correlation coefficient (R^2^ from 0.89–0.99). The constant K values for pesticide disappearance kinetics in soil ranged from 0.0116 to 0.1172. The DT_50_ values of fipronil and thiamethoxam ranged from 17.20–30.80 days and 5.92–59.95 days according to the experimental results, whereas they ranged from 10.42–30.58 days and 9.82–29.98 days according to the modeled data, respectively. The contour plot and response surface plot showed that the DT_50_ values of fipronil and thiamethoxam increased in alluvial soil and increased with low temperature. Also, the main factors affecting on the half-lives of fipronil and thiamethoxam were found to be temperature and sterilization. Published data demonstrate the likely degradation of fipronil and thiamethoxam in laboratory under different conditions. Further study for these pesticides is needed under field conditions to predict the dissipation behavior considering the expected climatic changes over all continents.

## Data Availability

The datasets used and/or analysed during the current study available from the corresponding author on reasonable request.
